# Mitigating role of thymoquinone rich fractions from *Nigella sativa* oil and its constituents, thymoquinone and limonene on lipidemic-oxidative injury in rats

**DOI:** 10.1186/2193-1801-3-316

**Published:** 2014-06-25

**Authors:** Shafeeque Ahmad, Zafarul H Beg

**Affiliations:** Department of Biochemistry, Jawahar Lal Nehru Medical College, Aligarh Muslim University, Aligarh, 202002 UP India

**Keywords:** *Nigella sativa*, Methanolic extract, Volatile oil, Thymoquinone, Limonene, Lipid peroxidation

## Abstract

Therapeutic role of *Nigella sativa* (NS) seed oil fractions, methanolic extract (ME) and volatile oil (VO) and their constituents, thymoquinone (TQ) and limonene (LMN) in relation to lipidemic-oxidative stress in Wistar rats was determined. The total phenolic contents of NS seed oil and their ME and VO extracts were 320.00 ± 3.00, 300.12 ± 0.04 and 288.41 ± 0.01 mg gallic acid equivalents per 100 g of NS oil, respectively. Their Fe^+2^ chelating activities were 870.00 ± 2.00, 222.31 ± 5.80 and 38.59 ± 1.43 mg EDTA equivalents per 100 g of NS oil, respectively. These fractions and compounds exhibited strong antioxidant activities against 2,2-diphenyl-1-picryl hydrazyl, 2,2-azinobis-3-ethylbenzothiazoline-6-sulfonic acid, nitric oxide and hydroxyl radicals. Potential antiperoxidative effects of these fractions and compounds were also observed in liposome, and lipidemic-induced lipid peroxidation in atherogenic suspension fed rats, pretreated with 100 mg ME, 20 mg VO, 10 mg pure TQ or 200 mg LMN for 30 days. ME containing ω-6 linoleic acid and palmitic acid natural compounds was highly effective against lipidemic oxidative stress than VO extract possessing thymol and isothymol phenolic natural antioxidant compounds. TQ, principal compound shared to both the extracts. The test fractions and compounds effectively reduced the erythrocyte and liver lipid peroxidation markers, conjugated diene, lipid hydroperoxide and malondialdehyde to near normal levels in the order ME > TQ > VO > LMN, by directly counteracting free radicals as well as suppressing hepatic HMG-CoA reductase activity. Our findings demonstrated that these natural products, preferably ME possess significant antioxidant activities, and may be recommended as new potential sources of natural antioxidants.

## Introduction

Hypercholesterolemia is highly correlated with atherosclerosis development and related cardiovascular diseases like coronary heart disease (Deepa and Varalakshmi 2005; Prasad and Kalra 1993). In comparison to cancer, these are the major source of morbidity and mortality in the developed countries (Stocker and Keaney 2004). It is well known that consumption of diet rich in cholesterol as well as saturated fat is the major factor for free radical production and lipid peroxidation, followed by oxidative stress and hypercholesterolemia (Bulur et al. 1995). Oxidative stress results from an imbalance between antioxidants and prooxidants (Sites and Mehlhorn 1986). Cell membranes are basically composed of lipids, especially polyunsaturated fatty acids. Such membranes are extremely susceptible to attack by free radicals, thereby causing cellular damage (Balkan et al. 2002; Cross et al. 1987). In comparison to liver, erythrocytes are highly susceptible to oxidative damage. The erythrocytes contain high concentrations of intracellular oxygen and hemoglobin (Hgb) (Cimen 2008). In addition, oxidative stress-induced hyperlipidemia followed by cholesterol and phospholipids accumulation in the erythrocytes, leads to further production of oxygen free radicals. Such free radicals are responsible for a number of changes like severe lipid peroxidation resulting in membrane damage and hemolysis (Sato et al. 1995; Niki et al. 1988).

It has been reported that the addition of one electron to O_2_ generates the superoxide anion radical (O_2_^**•**−^) (Halliwell and Gutteridge 2007). At least, there are two sources of O_2_^**•** −^ generation within erythrocytes: First, autoxidation of oxygenated hemoglobin (oxyHgb) (Giulivi and Daviess 1990) and second, the oxidation state of hemicrom iron (Fe^+3^) (Hebbel et al. 1982). This species is highly reactive and causes alterations in lipid and protein structure (Claster et al. 1984).


The above equation shows that dismutation of O_2_ will readily produce excessive amounts of H_2_O_2_. When H_2_O_2_ concentration increased, the increase in metHgb, lipid peroxidation, and spectrin-Hgb complexes are seen in a dose-dependent manner, resulting in cellular damage (Cimen 2008).


The most active reactive oxygen species (ROS), ^•^OH is produced, when H_2_O_2_ react with O_2_^**•** −^ and ferric or cupric ions. It is reported that ^•^OH is generated predominantly from H_2_O_2_ by Haber–Weiss reactions while the Fenton reaction is more important extracellularly (Cimen 2008; Al-Omar et al. 2004). Fenton chemistry catalyzed by transition metal is an excellent example of damaging organelles through free radical reactions (Halliwell and Gutteridge 2007). Excessive catabolism of feritin causes the labilization of lysosomal membrane, and this type of lysosomal damage might be consequence of iron-induced lipid peroxidation (Halliwell and Gutteridge 2007).

Different mechanisms have been attributed to explain antioxidant activity such as inhibition of chain initiation, peroxides decomposition, free radical scavenging, and binding of transition-metal ion catalysts (Mao et al. 2006). For the development of novel natural antioxidants, two factors are essentially utilized. The first is the consumer preference of natural antioxidants, because synthetic antioxidants, such as butylated hydroxytoluene and butylated hydroxyanisole have carcinogenic property (Onyeneho and Hettiarachchy 1992). The second factor is that polar antioxidants are more effective in nonpolar lipids, and vice versa (Frankel et al. 1996). This is called as polar paradox. Thus, various analytical methods and different substrates are employed to test antioxidant effectiveness.

Several epidemiological studies have been shown that plant foods, rich in phenolic compounds are protective against several deadly diseases such as cancer, neurodegenerative and cardiovascular diseases (Shahidi et al. 2011; Manach et al. 2005). A number of studies have shown that phenolic compounds possess antioxidant properties. Free radical scavenging properties of phenolic compounds depend upon their constituents and synergistic interactions (Shahidi and Naczk 2004). *Nigella sativa* (NS) has been shown that it possesses radical scavenging, antimicrobial and anti-inflammatory properties (Ramadan 2007; Ramadan et al. 2012). Thymoquinone (TQ), principal constituent of NS possesses antihistaminic, antibacterial, antihypertensive, hypoglycemic, anti-inflammatory and immunopotentiating activities (Kanter et al. 2006; Ali and Blunden 2003). TQ is also attributed to show antioxidant activity (Zafeer et al. 2012). Phenolic compounds, thymol and isothymol, also constituents of NS have been shown to possess antioxidant properties (Yanishlieva et al. 1999). The essential fatty acid, ω-6 linoleic acid (Spady et al. 1993), or ω-6 linoleic acid in the presence of palmitic acid has the hypolipidemic property (Champe et al. 2008). In our previous studies, hypolipidemic as well as antioxidant efficacies of methanolic extract (ME), volatile oil (VO) from NS seed oil and their constituents, TQ and limonene (LMN) were investigated, which effectively improved cardiometabolic risk parameters (Ahmad and Beg 2013a; Ahmad and Beg 2013b). However, a limited number of studies regarding NS seed oil, ME, VO, TQ and LMN have been reported. In the present study, total phenolic compounds of NS seed oil and its ME, VO extracts and their chelating activities against Fe^+2^ were determined. NS seed oil, ME, VO, their constituents TQ and LMN were also evaluated for their radical scavenging activities against 2,2-diphenyl-1-picryl hydrazyl, 2,2-azinobis-3-ethylbenzothiazoline-6-sulfonic acid, nitric oxide and hydroxyl radicals. In addition, the putative preventive effects of the test fractions and compounds on lipid peroxidation in erythrocytes and liver including H_2_O_2_-induced malondialdehyde release in the presence of lipidemic-oxidative stress, induced in rats by feeding with atherogenic suspension for 30 days, were investigated.

## Materials and methods

### Materials

Export quality edible *Nigella sativa* seed oil, was procured from a local store. Pure thymoquinone, limonene, 2,2-diphenyl-1-picryl hydrazyl, 2,2-azinobis-3-ethylbenzothiazoline-6-sulfonic acid, brain extract type VII and 1,1,3,3-tetramethoxypropane were purchased from Sigma-Aldrich Inc., USA, while thiobarbituric acid, 2-deoxy-2-ribose, sodium nitroprusside, sulphanilamide, napthylene diamine dihydrochloride, potassium nitrite, triphenyl phosphine and butylated hydroxyl toluene were purchased from HiMedia Laboratories Pvt. Ltd., Mumbai, India. Folin–Ciocalteu reagent was procured from Sisco Research Laboratories Pvt. Ltd., Mumbai, India. Sodium dodecyl sulfate was purchased from Bio-Rad Laboratories USA. Hemoglobin assay kit was purchased from Ranbaxy Diagnostics, New Delhi, India. Rat chow was procured from Ashirwad Industries, Chandigarh, India. Other chemicals and reagents used in this work were of analytical grade.

### Methanolic extract (ME) and volatile oil (VO) fractions from *Nigella sativa*seed oil

ME from NS seed oil was basically extracted as reported in our previous experiment (Ahmad and Beg 2013a). In brief, 10 g of NS seed oil was added to 100 ml of pure methanol and then stirred for 100 min at ambient temperature. Then this was kept at 45°C till the methanolic layer has evaporated completely. On the other hand, a procedure of steam distillation was used for the extraction of VO from NS seed oil. The procedure for VO extraction was employed essentially in the same method as used by Kanter et al. (2006) with minor modification. First of all, 12 g of NS seed oil was taken in a distillation flask, and then 400 ml of distilled water was added to it. The temperature of distillation unit was set to boiling point. Then 150 ml of the distillate containing VO was carefully collected in a dark glass bottle. For extraction of VO from the distillate, 50 ml of diethyl ether was added to it, and anhydrous sodium sulphate was used to remove moisture present in the sample. Thus diethyl ether extract containing VO was evaporated by keeping it at 40°C. The resulting ME and VO residues were flushed with nitrogen and kept in dark colored bottles at 4°C.

### Total phenolic contents (TPC) in NS oil, and its ME and VO

With slight modification, total phenolic contents of the NS seed oil and its ME and VO fractions were determined in triplicate by using the Folin–Ciocalteu reagent (Yu et al. 2002a). The reaction mixture contained several concentrations of NS oil, ME or VO extracts in DMSO-saline, 125 μl of the Folin–Ciocalteu reagent, and 375 μl of 20% sodium carbonate in a total volume of 2.5 ml. After 2 h of incubation at ambient temperature, the absorbance at 765 nm was measured. The TPC was calculated by using gallic acid as standard.

### Free radical scavenging activities

#### 2,2-Diphenyl-1-picryl hydrazyl (DPPH^•^) scavenging activity

With minor modification, 2,2-diphenyl-1-picryl hydrazyl scavenging activities of NS seed oil, ME, VO fractions, pure TQ and LMN were determined in triplicate by the method of Mensor et al. (2001). The reaction mixture contained several concentrations of NS seed oil, ME, VO fractions or pure TQ in methanol, whereas LMN was dissolved in Tween 80-PBS. The reaction was started by the addition of freshly prepared methanolic solution of DPPH^•^ in a total volume of 3.5 ml, mixed thoroughly and allowed to react in dark at ambient temperature. After 30 min the absorbance of sample at 518 nm was read. The concentration dependency of the above antioxidant fractions and compounds was done by plotting the percent of DPPH^*•*^ remaining against each level of antioxidants by using standard DPPH^*•*^. The percent antioxidant activity of the above antioxidants was calculated according to the following formula:


#### Hydroxyl radical (^•^OH) scavenging activity

The scavenging of OH radical for the test drugs was done by the method of Halliwell et al. (1987). Briefly, one ml of the reaction mixture contained 2.8 mM 2-deoxy-2-ribose dissolved in 200 mM of KHPO_4_–K_2_HPO_4_ buffer pH 7.4, and 20 μM FeCl_3_ and 0.104 mM EDTA (1:1 v/v), 1.0 mM H_2_O_2_, 1.0 mM ascorbic acid and several concentrations of NS seed oil, ME, VO, pure TQ or LMN dissolved in Tween 80-PBS. The reaction mixture was mixed and incubated for 1 h at 37°C, followed by the addition of 1.0 ml of 1% thiobarbituric acid prepared in 50 mM NaOH and 1.0 ml of 2.8% TCA. The samples were then boiled for 20 min at 100°C, cooled to room temperature and the absorbance was recorded at 532 nm against a reagent blank in a Beckman DU 640 spectrophotometer. A control blank without test fractions or compounds was used to determine the percentage inhibition of deoxyribose degradation by the above test fractions or compounds_._

#### 2,2-Azinobis-3-ethylbenzothiazoline-6-sulfonic acid (ABTS^•+^) scavenging activity

With slight change, the concentration-dependent scavenging efficiencies of NS seed oil, ME, VO, TQ and LMN against ABTS^•+^ were evaluated by the method of Re et al. (1999), where ABTS radical cation was produced by mixing equal volumes of 7 mM ABTS and 2.45 mM potassium persulfate solutions and allowed them to react for 12 h at ambient temperature in the dark. The reaction mixture in triplicate contained several concentrations of NS seed oil, ME, VO or pure TQ dissolved in methanol, whereas LMN was dissolved in Tween 80 and 10 mM phosphate buffer saline pH 7.4 and 500 μl of 60-fold diluted methanolic solution of ABTS^•+^ in a total volume of 1.1 ml. The samples were mixed thoroughly and allowed to react in dark at room temperature. The absorbance was taken at 734 nm after 7 min against methanol or distilled water using the Beckman DU 640 spectrophotometer. A control blank lacking test fractions or compounds, was used to calculate their percent ABTS^•+^ scavenging capacities.

#### Nitric oxide (NO^•^) scavenging activity

Nitric oxide scavenging activities of several concentrations of NS seed oil, ME, VO, TQ and LMN were determined according to the procedure of Marcocci et al. (1994). When sodium nitroprusside is allowed to dissolve in aqueous medium nitric oxide is spontaneously generated from it at physiological pH which interacts with oxygen to produce nitrite ions that can be estimated by the use of Greiss reagent. Scavengers (antioxidants) of nitric oxide compete with oxygen leading to reduced production of nitric oxide. One ml of the reaction mixture contained 500 μl of various concentrations of the samples dissolved in DMSO-saline was mixed with 5 mM sodium nitroprusside prepared in 10 mM potassium phosphate buffer pH 7.4 then incubated at 25°C for 150 min. At the end of the incubation, the samples from the above were allowed to react with 1 ml of Greiss reagent containing equal volume of solutions A (2% sulfanilamide and 4% H_3_PO_4_) and B (0.2% naphthylethylenediamine dihydrochloride). The absorbance of the chromophore formed during the diazotization of nitrite with sulfanilamide and subsequent coupling with naphthylethylenediamine was read at 542 nm in the Beckman DU 640 spectrophotometer. Their percent NO^•^ scavenging capacities were calculated by using standard potassium nitrite.

#### Chelating activity

The concentration-dependent ferrous (Fe^+2^) chelating capacities of NS seed oil, ME and VO extracts were determined as described by Yu et al. (2002b). The reaction mixture contained 46.296 μM FeSO, 250 μl of different concentrations of the samples dissolved in 1% SDS, 3.7037 mM Tris–HCl buffer pH 7.4, 1.185 mM 2,2’-bipyridyl prepared in 37.037 mM hydrochloride and 106.592 mM hydroxylamine hydrochloride in the total volume 5.4 ml, was made by adding 2.5 ml of ethanol. A control blank without test samples was conducted in an identical manner. The absorbance of sample was read at 522 nm against distilled water and used to calculate Fe^+2^ chelating capacity using a standard curve prepared with EDTA.

#### Nonenzymatic lipid peroxidation

The inhibition of nonenzymatic lipid peroxidation in phospholipid liposomes, prepared from type VII Folch bovine brain extract, by four concentrations of ME, VO or TQ, was carried out essentially the same as previously reported method of Houghton et al. (1995). Phospholipid liposomes were prepared from brain extract Type VII from bovine brain by mixing with 200 mM of KHPO_4_–K_2_HPO_4_ buffer pH 7.4 (5 mg/mL) and stored at 4°C for 6 days. It was then sonicated under cooling with ice until a milky solution was obtained. One ml of the reaction mixture contained 500 μl of phopholipid suspension, 3 mM sodium phosphate buffer saline pH 7.4 containing different concentrations of the test samples dissolved in Tween 80 and PBS, 1 mM FeCl_3_ and 1 mM ascorbic acid to start peroxidation. The reaction mixture was mixed and incubated for 1 h at 37°C. At the end of incubation, 1.0 ml of 1% TBA prepared in 50 mM NaOH, 1.0 ml of 2.8% TCA and 0.1 ml of 2% BHT prepared in ethanol were added to the reaction mixture. The samples were boiled for 20 min at 100°C, cooled to room temperature then 2.5 ml of n-butanol was added. The reaction mixtures were then centrifuged at 3500 rpm for 5 min. The absorbance was recorded at 532 nm against a reagent blank in the Beckman DU 640 spectrophotometer. All reagents were prepared freshly. A control blank without test samples was conducted in an identical manner. The inhibition of malondialdehyde (MDA) formation by the test fractions and compound was represented as a percentage of the control (minus antioxidant) value.

### Animals and treatments

Approval of this experimental study in animals was obtained from the Board of Studies of Biochemistry department and Ethics Committee of Jawahar Lal Nehru Medical College, A.M.U. Healthy male Wistar rats and their weights 180–210 g, from inbred colony maintained by the central animal facility of Jawahar Lal Nehru Medical College, were used. Standard rat chow and water were available for these animals ad libitum. The test fractions, 10% ME, 2% VO from NS seed oil, and the compounds 1% TQ and 20% LMN suspensions for the treatment of rats were prepared according to Kanter (2008); Altan et al. (2007); El Gazzar et al. (2006) with slight change by dissolving in DMSO (12.5% final concentration) and finally homogenized with saline. To induce hyperlipidemia, an atherogenic suspension consisted of (w/v) 0.5% cholesterol, 3% coconut oil and 0.25% cholic acid that is 5 mg cholesterol, 30 mg coconut oil and 2.5 mg cholic acid per ml was prepared by mixing in a Potter-Elvehjem homogenizer. Rats were randomly assigned into the following different treatment groups:

- Normolipidemic control (NLP-C): This normal control group containing five albino rats was orally administered one ml of saline containing 12.5% DMSO twice per day.
- Hyperlipidemic control (HLP-C): The four rats in hyperlipidemic control group were orally given 0.5 ml of saline containing 12.5% DMSO, before the administration of 0.5 ml of the above prepared atherogenic suspension twice per day, with no drug intervention.
- Hyperlipidemic ME (HLP-ME): Before administration of atherogenic suspension, four rats in this treated group received one ml of the saline suspension containing 100 mg of ME orally in two equal doses (morning and evening) of 0.5 ml each, for 30 days.
- Hyperlipidemic VO (HLP-VO): Before administration of atherogenic suspension, four rats in this treated group received one ml of the saline suspension containing 20 mg of VO orally in two equal doses (morning and evening) of 0.5 ml each, for 30 days.
- Hyperlipidemic TQ (HLP-TQ): Before administration of atherogenic suspension, four rats in this treated group received one ml of the saline suspension containing 10 mg of TQ orally in two equal doses (morning and evening) of 0.5 ml each, for 30 days.
- Hyperlipidemic LMN (HLP-LMN): Before administration of atherogenic suspension, four rats in this treated group received one ml of the saline suspension containing 200 mg of LMN orally in two equal doses (morning and evening) of 0.5 ml each, for 30 days.

### Blood collection and erythrocyte preparation

At the end of treatment, blood was drawn from cardiac puncture of anaesthetized, and overnight fasted rats in each group and collected in heparinised tubes. And it was mixed gently by inversion 2–3 times and incubated at 4°C for 2–3 h. Centrifugation of blood was performed at 2,500 rpm for 30 min to separate plasma and buffy coat. The packed erythrocytes from blood thus, obtained were resuspended in physiological saline and centrifuged again at 1,500 rpm for 10 min at 4°C, and this procedure was done two times more. The procedure described by Lakshmi and Rajagopal (1998) was employed for hemolysate preparation from a portion of packed erythrocytes.

### Preparation of liver homogenate

Livers from each rat were blotted. And the livers of each group were cut into small pieces. With the help of a waring blender, 10 g of wet tissue was homogenized with 90 ml of chilled 0.1 M sodium phosphate buffer, pH 7.4, containing 1.17% KCl. The homogenate was centrifuged at 1,000 rpm for 10 min at 4°C. A portion of the homogenate obtained from liver samples in each group was aliquoted and stored at −20°C for future use. Other necessary steps and precautions were taken for the sample preparation and its storage.

### Determination of hydrogen peroxide-induced MDA release

For the determination of MDA released in intact erythrocytes, the procedure of Cynamon et al. (1985) was employed*.* On the other hand, the determination of basal MDA content in erythrocyte hydrolysate was performed according to the method of Stocks and Dormandy (1971). The concentrations of MDA in these samples were calculated by taking a standard malondialdehyde (Liu et al. 1982).

### Determination of conjugated diene (CD), lipid hydroperoxide (LOOH) and MDA in liver

Lipid contents were extracted from liver, according to the method of Folch et al. (1957). In Beckman DU 640 spectrophotometer, the absorbance of lipid residues dissolved in 1.5 ml of cyclohexane was taken at 234 nm against a cyclohexane blank, and their CD concentration was determined by using a molar extinction coefficient of 2.52 × 10^4^ M^−1^ cm^−1^. The method of Nourooz-Zadeh et al. (1996) for LOOH quantification from liver homogenates was used, and hydroperoxide contents were determined by using a molar absorption coefficient of 4.3 × 10^4^ M^−1^ cm^−1^. The method of Ohkawa et al. (1979) was used for the determination of lipid peroxides in liver homogenate, and malondialdehyde was used to quantify MDA concentration (Liu et al. 1982).

### Protein estimation

The method of Bradford (1976) was used to quantify protein content present in liver homogenate by using bovine serum albumin as standard. Liver homogenates were first precipitated with 10% TCA, and then the protein pellets were dissolved in 0.5 N NaOH. Suitable aliquots of the animal samples then were used for protein determination.

### Statistical evaluation

For statistical analysis of the data, one way analysis of variance was used, then Tukey’s Kramer multiple comparison test was employed. For statistical determination, P values < 0.05 were considered as significant.

Note: In treated groups of animals, the efficacy of test samples was expressed in terms of reduction in percent or it may be called as percent restoration value in relation to reduction. It can be calculated using the below equation:


## Results

### Yield of ME and VO, their total TPC and Fe^+2^- chelating activities

**Figure 1:**
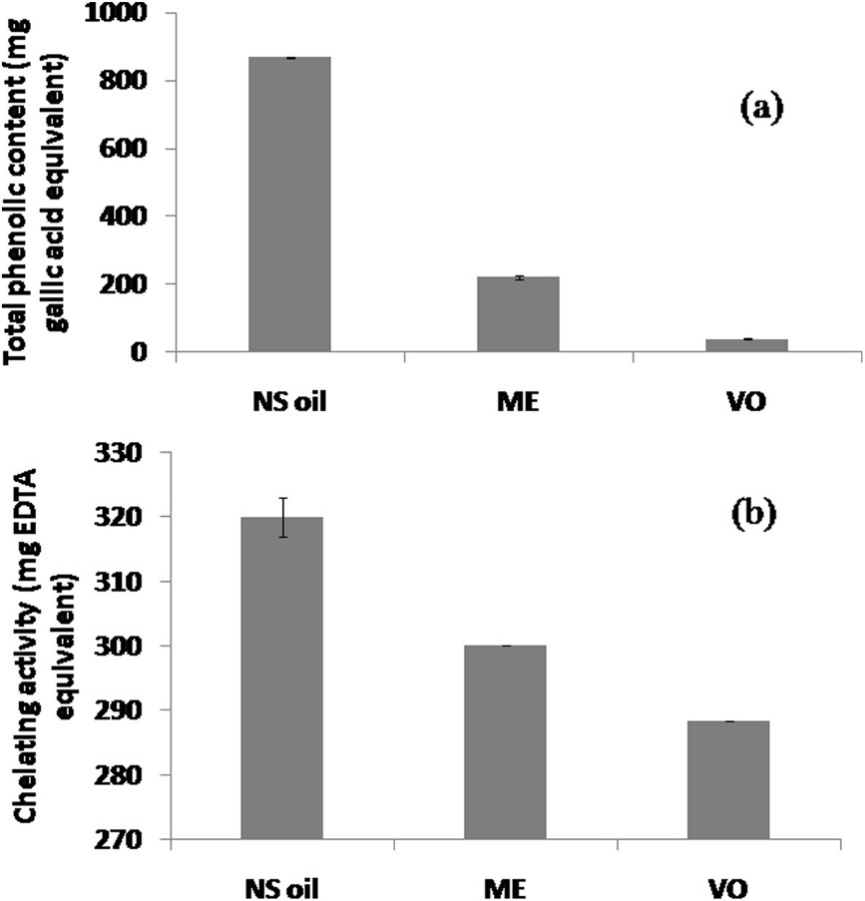
**Total phenolic contents (TPC) and Fe** ^**2+**^ **-chelating activities of** ***Nigella sativa*** **(NS) seed oil and its methanolic extract (ME) and volatile oil (VO), and their values are mean ± SD from 3 to 8 different experiments done in triplicate.** Panel **(a)**: TPC of NS oil and its fractions are expressed as mg gallic acid equivalent per 100 g of NS oil; or per 21.49 g of ME (average yield per 100 g of NS oil from 3 extractions); or per 1.170 g VO (average percent yield from 4 preparations). Panel **(b)**: Chelating activities of NS oil and its fractions are expressed as mg EDTA equivalent per 100 g of NS oil; or per 21.49 g of ME (average yield per 100 g of NS oil from 3 extractions); or per 1.170 g VO (average percent yield from 4 preparations).

An average yield of ME was 20.380 ± 0.025% from 40 extractions of NS seed oil with pure methanol. The average yield of VO was 1.024 ± 0.007% from 23 extractions of NS seed oil with steam distillation. The average TPC was 320.00 ± 3.00 mg gallic acid equivalents (GE) in 100 g of NS seed oil. While the average yield of TPC from 3 ME or 4 VO extracts was 300.12 ± 0.04 and 288.41 ± 0.01 mg GE/21.49 g ME or per 1.170 g of VO, respectively (Figure 1a). A significant chelating activity against Fe^+2^ in NS seed oil and its ME and VO extracts was seen. Oil from NS seeds exhibited a value of 870 mg EDTA equivalent per 100 g oil, whereas in ME or VO the chelating capacity was 222 or 39 mg EDTA equivalent per 21.49 g of ME or per 1.170 g VO isolated from 100 g of NS seed oil (Figure 1b).

### Comparison of scavenging activities of NS Seed oil and its ME and VO extracts; pure TQ and LMN against DPPH^•^, ^•^OH, ABTS^•+^ and NO^•^

Data in Table 1 represent the comparison of scavenging activities of NS seed oil, and its ME and VO extracts with pure TQ, constituent of ME and VO, against DPPH^**•**^, ^•^OH, ABTS^•+^ and NO^•^. The ME had the strongest DPPH^**•**^ scavenging capacity, with an IC_50_ of 42 μg/ml, followed by the TQ (57 μg/ml) and VO (88 μg/ml), respectively, while NS seed oil had an IC_50_ value of 5 mg/ml. Similar to DPPH^**•**^ scavenging capacities among the five test fractions and compounds, the order of quenching efficiencies of OH radicals were ME > TQ > VO > NS oil > LMN (here value of LMN has been not shown). The IC_50_ values for these antioxidants were 140, 186, 290 μg/ml and 15 mg/ml, respectively. The NS seed oil and its ME and VO extracts, TQ and LMN were also evaluated for their scavenging activity against the ABTS^•+^, generated through potassium persulfate mediated oxidation reaction (Table 1). The NS seed oil, ME and VO extracts as well as TQ directly reacted with and quenched ABTS^•+^, with an IC_50_ of 200, 8, 17 and 11 μg/ml, respectively. Consistent with these results, incubation of above five test fractions and compounds caused a concentration dependent inhibition of NO^•^ generation during in vitro incubation. The 50% inhibition of NO^•^ generation for NS seed oil was 8 mg/ml, while for ME and VO fractions of NS seed oil as well as TQ the values were 69, 140 and 90 μg/ml.

**Table 1:** **Comparison of scavenging activities of NS oil and its ME and VO fractions and pure TQ against 2,2-diphenyl-1-picryl hydrazyl (DPPH), hydroxyl (OH), 2,2-azinobis-3-ethylbenzothiazoline-6-sulfonic acid (ABTS) and nitric oxide (NO) radicals**

Type of free radicals	Test fractions or compounds			
	NS oil	ME	VO	TQ
	IC _50_^#^ (μg or mg ^**^/ml)			
A. DPPH^•^	5.12 ± 0.03^*^	42.14 ± 0.0^*^	88.39 ± 0.04^*^	57.15 ± 0.05^*^
	(1.42-10.0)^**^	(7.14-142.85)	(14.28-342.85)	(8.57-171.42)
B. ^•^OH	14.91 ± 0.01	140.0 ± 0.53	290.0 ± 0.27	186.0 ± 0.5
	(5.0-35.0)^**^	(25.0-500.0)	(50.0-1000.0)	(25.0-500.0)
C. ABTS^•+^	200.0 ± 0.83	7.81 ± 0.11	17.36 ± 0.17	10.68 ± 0.07
	(45.45-545.45)	(1.81-27.27)	(4.54-54.54)	(2.72-22.72)
D. NO^•^	7.60 ± 0.01	68.75 ± 0.24	140.0 ± 0.22	89.75 ± 0.25
	(1.0-20.0)^**^	(5.0-250.0)	(25.0-500.0)	(5.0-250.0)

^#^IC_50_ is defined as the concentration (μg or mg^**^/ml; with range in parenthesis) of test fractions or compounds sufficient to quench or inhibit 50% of the above free radicals (A-D) under standard experimental conditions.

^*^Values are mean ± SD, of triplicate determinations, were calculated from a concentration dependent plot of each test fraction or compound versus the percent of antioxidant activity.

### Inhibition of nonenzymatic lipid peroxidation

**Figure 2:**
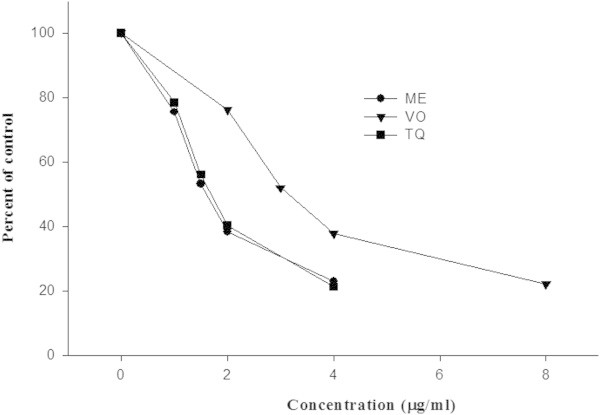
**Inhibition of nonenzymatic lipid peroxidation in phospholipid liposomes by ME and VO fractions and pure TQ.** Lipid peroxidation in phospholipid liposomes, prepared from type VII Folch bovine brain extract (Sigma-Aldrich Inc. USA), was induced by Fe^3+^-ascorbate. Lipid peroxidation was monitored by measuring the formation of malondialdehye. MDA value in control samples incubated without ME, VO or TQ is represented as 100%, whereas inhibition of lipid peroxidation at indicated concentrations of the above fractions and TQ are represented as a percentage of the control value. MDA values represent mean of triplicate determinations.

Nonenzymatic lipid peroxidation can clearly be observed when ox brain phospholipid liposomes incubated with FeCl_3_ and ascorbic acid. In the presence of reducing agent such as ascorbic acid, Fe^+3^ produces OH radical. As shown in Figure 2, the IC_50_ for TQ was 1.72 μg/ml, whereas for ME and VO fractions, these values were 1.62 and 3.15 μg/ml, indicating a ~ 2-fold higher inhibition of lipid peroxidation by ME and TQ than VO extract.

### Effect on membrane lipid peroxidation in erythrocytes

**Figure 3:**
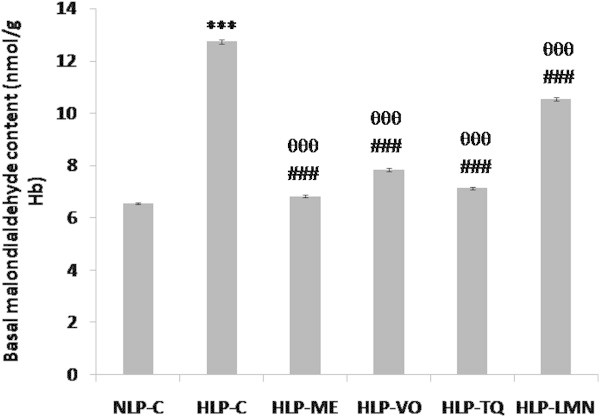
**Effect of methanolic extract (ME), volatile oil (VO) fractions, and their constituents, thymoquinone (TQ) and limonene (LMN) on the basal malondialdehyde (MDA) content in erythrocyte hemolysates of atherogenic suspension fed rats.** NLP-C, normolipidemic control (n = 5); HLP-C, hyperlipidemic control ((n = 4)) given one ml of suspension containing 5 mg cholesterol, 30 mg coconut oil and 2.5 mg cholic acid/rat/day; whereas four rats in HLP-ME, HLP-VO, HLP-TQ and HLP-LMN groups were fed one ml of 100 mg ME, 20 mg VO, 10 mg TQ or 200 mg LMN prior to administration of the above atherogenic suspension/rat/day for 30 days of duration. Values are mean ± SD from erythrocyte hemolysates of animals (n = 4 in all groups except n = 5 in NLP-C group). The value of HLP-C control group was statistically significant from NLP-C control group (***p < 0.001). The values of treated groups, HLP-ME, HLP-VO, HLP-TQ and HLP-LMN were significantly different from HLP-C (^###^p < 0.001), and NLP-C groups (^θθθ^p < 0.001).

**Figure 4:**
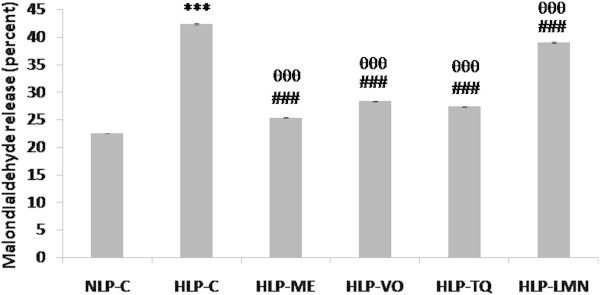
**Effect of methanolic extract (ME), volatile oil (VO) fractions, and their constituents, thymoquinone (TQ) and limonene (LMN) on the H**_**2**_ **O**_**2**_ **-induced malondialdehyde (MDA) in intact erythrocytes of atherogenic suspension fed rats.** NLP-C, normolipidemic control; HLP-C, hyperlipidemic control given one ml of suspension containing 5 mg cholesterol, 30 mg coconut oil and 2.5 mg cholic acid/rat/day; whereas rats in HLP-ME, HLP-VO, HLP-TQ and HLP-LMN were fed one ml of 100 mg ME, 20 mg VO, 10 mg TQ or 200 mg LMN prior to administration of the above atherogenic suspension/rat/day for 30 days of duration. Values are mean ± SD from intact erythrocytes of each animal (n = 4 in all groups except n = 5 in NLP-C group). The value of HLP-C control group was statistically significant from NLP-C control group (***p < 0.001). The values of treated groups, HLP-ME, HLP-VO, HLP-TQ and HLP-LMN were significantly different from HLP-C (^###^p < 0.001), and NLP-C groups (^θθθ^p < 0.001).

As shown in Figure 3, ex vivo MDA content of erythrocyte hemolysate was increased (94%) from 6.56 in NLP-C to 12.75 nmole/gHb in HLP-C. In rats treated with ME, VO or TQ, 94% increase in MDA was significantly blocked and reduced to 96%, 84% and 92%, respectively, of NLP-C value, while in HLP-LMN this reduction in MDA was 62%. Intact erythrocytes from HLP-C stressed rats showed a greater susceptibility to H_2_O_2_-induced lipid peroxidation than those from NLP-C group. An increase of 88% (p < 0.001) in release of MDA in HLP-C was seen in comparison to NLP-C value. This increase in the formation of MDA was significantly reduced to a level which was 89%, 80%, 82% and 58% of control value in NLP-C, after 30-day treatment of atherogenic suspension fed rats with ME, VO, TQ or LMN, respectively (Figure 4).

### Impact on liver lipid peroxidation products

**Figure 5:**
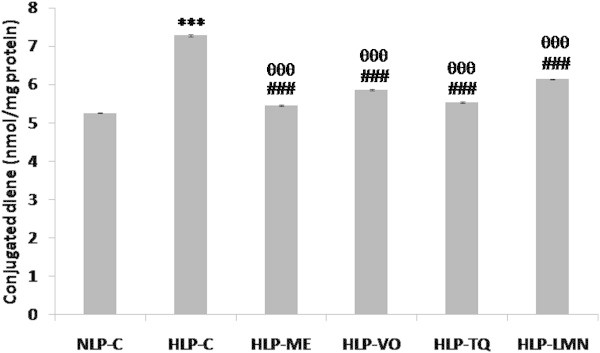
**Effect of methanolic extract (ME) and volatile oil (VO) extracts of** ***Nigella sativa*** **(NS) oil, thymoquinone (TQ) and limonene (LMN) on liver conjugated diene (CD) concentration in atherogenic suspension fed rats.** NLP-C, normolipidemic control; HLP-C, hyperlipidemic control given one ml of suspension containing 5 mg cholesterol, 30 mg coconut oil and 2.5 mg cholic acid/rat/day; whereas rats in HLP-ME, HLP-VO, HLP-TQ and HLP-LMN were fed one ml of 100 mg ME, 20 mg VO, 10 mg TQ or 200 mg LMN prior to administration of the above atherogenic suspension/rat/day for 30 days of duration. Values are mean (nmol/mg protein; CD values are expressed as nmol MDA equivalents) ± SD from homogenates of each animal (n = 4 in all groups except n = 5 in NLP-C group). The value of HLP-C control group was statistically significant from NLP-C control group (***p < 0.001). The values of treated groups, HLP-ME, HLP-VO, HLP-TQ and HLP-LMN were significantly different from HLP-C (^###^p < 0.001), and NLP-C groups (^θθθ^p < 0.001).

**Figure 6:**
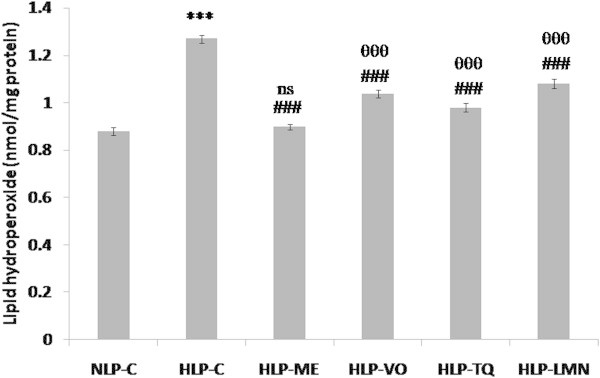
**Effect of methanolic extract (ME) and volatile oil (VO) extracts of** ***Nigella sativa*** **(NS) oil, thymoquinone (TQ) and limonene (LMN) on hepatic lipid hydroperoxide (LOOH) concentration in atherogenic suspension fed rats.** NLP-C, normolipidemic control; HLP-C, hyperlipidemic control given one ml of suspension containing 5 mg cholesterol, 30 mg coconut oil and 2.5 mg cholic acid/rat/day; whereas rats in HLP-ME, HLP-VO, HLP-TQ and HLP-LMN were fed one ml of 100 mg ME, 20 mg VO, 10 mg TQ or 200 mg LMN prior to administration of the above atherogenic suspension/rat/day for 30 days of duration. Values are mean (nmol/mg protein) ± SD from homogenates of liver samples in each animal (n = 4 in all groups except n = 5 in NLP-C group). The value of HLP-C control group was statistically significant from NLP-C control group (***p < 0.001). The values of treated groups, HLP-ME, HLP-VO, HLP-TQ and HLP-LMN were significantly different from HLP-C (^###^p < 0.001) group. The values of treated HLP-VO, HLP-TQ and HLP-LMN groups were significantly different from NLP-C group (^θθθ^p < 0.001), while the value of treated HLP-ME group was not found significantly different from NLP-C group (ns in the figure refers to not significant).

**Figure 7:**
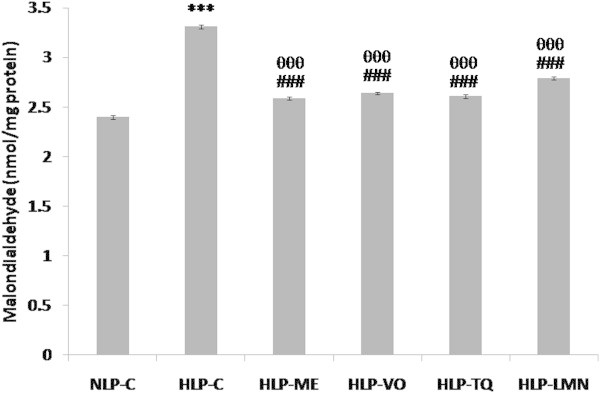
**Effect of methanolic extract (ME) and volatile oil (VO) extracts of** ***Nigella sativa*** **(NS) oil, thymoquinone (TQ) and limonene (LMN) on liver malondialdehyde (MDA) concentration in atherogenic suspension fed rats.** NLP-C, normolipidemic control; HLP-C, hyperlipidemic control given one ml of suspension containing 5 mg cholesterol, 30 mg coconut oil and 2.5 mg cholic acid/rat/day; whereas rats in HLP-ME, HLP-VO, HLP-TQ and HLP-LMN were fed one ml of 100 mg ME, 20 mg VO, 10 mg TQ or 200 mg LMN prior to administration of the above atherogenic suspension/rat/day for 30 days of duration. Values are mean (nmol/mg protein) ± SD from homogenates of liver samples in each animal (n = 4 in all groups except n = 5 in NLP-C group). The value of HLP-C control group was statistically significant from NLP-C control group (***p < 0.001). The values of treated groups, HLP-ME, HLP-VO, HLP-TQ and HLP-LMN were significantly different from HLP-C (^###^p < 0.001), and NLP-C groups (^θθθ^p < 0.001).

As depicted in Figures 5, 6 and 7, CD, LOOH and MDA formation in liver were increased from 5.26, 0.88 and 2.40 in NLP-C to 7.28 (38%), 1.27 (44%) and 3.31 (38%) nmol/mg protein, respectively, in HLP-C rats. Pretreatment of atherogenic suspension fed rats with test fractions and compounds blocked this increase in CD formation and restored them to a value which was 97% in HLP-ME, 90% in HLP-VO, 95% in HLP-TQ and 86% in HLP-LMN of corresponding normal values in NLP-C (Figure 5). The value of LOOH product, the increased value was significantly reduced to 0.899, 1.039, 0.980 and 1.081 nmol/mg protein in HLP-ME, HLP-VO, HLP-TQ and HLP-LMN, respectively when compared to NLP-C rats (Figure 6). In comparison to HLP-C rats, pretreatment with the test fractions and pure compounds the MDA values were also decreased by 93%, 91%, 92% and 86% in ME, VO, TQ and LMN treated groups, respectively (Figure 7).

## Discussion

Natural antioxidant compounds that can protect tissues from free radical attack are considered essential in terms of inhibiting oxidative damage. Thus, there is highly increasing interest in the consumption of plant products rich in phenolic compounds. These natural compounds possessing antioxidant actions contribute to protection from oxidative stress-induced pathogenesis of disease. Accordingly, the present study was initiated to investigate both in vitro and in vivo antioxidative efficacies of ME and VO extracts from NS seed oil, pure TQ as well as LMN, in rats fed an atherogenic suspension for 30 days. ME and VO fractions contain different types of phenolic as well as terpenoid compounds including other pharmaceutical compounds as identified by their GC-MS analysis (Table 2). The presence of good quantity of phenolic as well as fatty acids compounds in NS seed oil, ME and VO fractions (Table 2, Figure 1, panel a) may provide overall antioxidant activities. These results suggest that NS seed and/or oil may serve as a dietary source of phenolic compounds, which may play a role in disease prevention through improved nutrition. Initiation of the oxidative chain reactions may be facilitated by transition metals that act as catalysts through the promotion of the generation of the first few radicals (Nawar 1996). Content of transition metals may be reduced by chelating agents, such as EDTA, and consequently improve human health and food stability via inhibition of radical-mediated oxidative chain reactions in biological or food systems. Our results also demonstrate a significant chelating activity against Fe^+2^ in the NS seed oil and its ME and VO extracts (Figure 1, panel b).

**Table 2:** **Major chemical composition of methanolic extract (ME) and volatile oil (VO) extracted from** ***Nigella sativa*** **seed oil**

Serial no.	ME compounds	VO compounds
1	Limonene	p-Cymene
2	Thymoquinone	(+)-trans, trans-5-Caranol
3	Butylated hydroxytoluene	4-Terpineol
4	Palmitic acid	p-Cymen-8-ol
5	Citronellyl butyrate	Thymoquinone
6	Phytol	Thymol
7	Linoleic acid	Isothymol
8	Silane, [1-(5-ethenyltetrahydro-5-methyl-2-furanyl)-1-methylethoxy]trimethyl-, trans-	Copaene
9	Linoleic acid trimethylsilyl esterI	Limonene oxide
10	Glycerol 1-monolinolate	Longifolene-(V4)
11	-	p-tert-Butylpyrocatechol
12	-	Rimuen
13	-	Cephrol
14	-	Citronellal

For complete chemical constituents of ME and VO extracts as their compounds identified by GC-MS analysis, can be seen in our research article, investigated by Ahmad and Beg (2013a).

In general, two or more radical systems are employed for testing efficacies of antioxidants. Therefore, it is better to consider different assays for determining the strength of suspected antioxidant activity. Since 94% and 90% of total phenolic compounds present in NS oil were recovered in phenolic compounds rich ME and VO extracts (Figure 1, panel a). Their antioxidant activities were estimated in terms of hydrogen donating or radical scavenging capacities against DPPH^**•**^**,**^•^OH, ABTS^•+^ and NO^•^, and compared scavenging activities of TQ and LMN, two constituents of VO fraction of NS oil. It is noteworthy that among the test fractions and compounds, ME had the strongest scavenging activity with an IC_50_ value of 8 μg/ml against the ABTS^•+^ generated through a chemical oxidation reaction, followed by TQ (11 μg/ml), VO extract (17 μg/ml) and NS seed oil (200 μg/ml), respectively. The results show that radical scavenging efficiencies of ME against DPPH^•^**,**^•^OH, ABTS^•+^ and NO^•^ are on the average stronger by 108% and 34%, when compared to corresponding radical quenching efficacies of VO and TQ.

Nonenzymatic lipid peroxidation can clearly be noticed when ox brain phospholipid liposomes were incubated with FeCl_3_ and ascorbic acid. In the presence of reducing agent such as ascorbic acid, Fe^+3^ produces OH radical (Burits and Bucar 2000). Estimation of the pink pigment, lipid peroxidation (Aruoma 1996) is widely accepted, and it is generated through reaction of 2-thiobarbituric acid with malondialdehyde (MDA) (Kosugi et al. 1987). Figure 2 represents a concentration dependent (0–8 μg/ml) OH radical scavenging effect of ME and VO extracts from NS seed oil and TQ, which resulted in a strong inhibition of nonenzymatic lipid peroxidation in phospholipid liposomes by reducing the formation of MDA. These results are clearly supported by plasma ^•^OH scavenging property of thymoquinone rich fraction (TQRF) and TQ (Ismail et al. 2010). The data revealed that ME exhibited a profound antioxidant effect and only at 1.62 μg/ml, it could inhibit 50% lipid damage, followed by TQ and VO extract with IC_50_ values of 1.72 and 3.15 μg/ml, respectively. These results are consistent with the data in Table 1, where ME exhibited a 2-fold higher radical scavenging activities compared to VO extract. The combined results presented in Table 1 and Figure 2, established the order of scavenging efficiencies (IC_50_ values) against the above four free radicals was ME > TQ > VO > NS seed oil > LMN, however, LMN exhibited a highly poor radical scavenging/antioxidant capacity. The ME exhibited a two-fold greater antioxidant capacity in comparison to VO extract, which is apparently due to the presence of the antioxidant compounds as well as additional mixture of phenolic compounds as explored by their GC-MS analysis (Table 2). Houghton et al. (1995) have reported that the fixed oil of the NS seed possessed greater antioxidant capacity than TQ. Thus, ME enriched with the radical scavenging agents plus good amount of mixture of phenolic compounds, which evidently contributed independently or synergistically with TQ to substantially enhance its antioxidant efficiency.

In vivo investigations revealed that feeding of an atherogenic suspension to rats containing 5 mg cholesterol, 30 mg coconut oil and 2.5 mg cholic acid/rat/day for 30 days was associated with increased ROS/free radicals production, lipid peroxidation and oxidative stress in erythrocytes and liver. The results in Figures 3, 4, 5, 6 and 7 show that due to severe lipidemic-oxidative stress, significant increments in conjugated diene (CD), lipid hydroperoxide (LOOH) and/or MDA/TBARS levels, markers of endogenous lipid peroxidation, were observed in the erythrocytes and liver of untreated HLP-C animals. In addition, intact erythrocytes from these rats showed a further increase in susceptibility to H_2_O_2_-induced MDA release when compared to basal MDA levels (Figure 4). Furthermore, in these rats, 60% higher formation of MDA, the end product of lipid peroxidation, in erythrocytes was seen, compared to ex vivo MDA levels in liver, indicating a much severe oxidative damage in these stressed erythrocytes. Oral feeding of 100 mg ME and 20 mg VO extracts, 10 mg pure TQ or 200 mg LMN to rats, effectively reduced the erythrocyte and liver ex vivo CD, LOOH and MDA levels including H_2_O_2_-induced MDA release in intact erythrocytes. These findings are in full agreement with in vitro results of the present study, where phenolic compounds rich ME of NS oil exhibited a 2-fold higher inhibition of nonenzymatic lipid peroxidation in phospholipid liposomes by reducing the formation of MDA, through OH radical-scavenging activity, when compared to VO fraction. Thus, our discussion particularly supports the investigation done by Ismail et al. (2010). They reported that treatment of 1% cholesterol enriched diet fed rats with TQRF from NS seeds and TQ significantly increased the plasma hydroxyl radical scavenging activity. The antiperoxidative/radical scavenging efficacies of test fractions and compounds in erythrocytes and liver was in the order ME > TQ > VO > LMN.

The antioxidative or antiperoxidative properties of ME, VO and their fractions TQ and LMN in rats are associated with multiple mechanisms. As we are well aware of atherogenic suspension that induces oxidative stress and increases the susceptibility of membrane lipids to oxidation. Oxidative stress results from deviation from equilibrium between antioxidant and oxidant systems. HMG-CoA reductase an important rate-limiting enzyme regulates cholesterol level. As in our previous experiments, administration of ME, VO and their TQ and LMN fractions in atherogenic suspension fed rats, the activity of HMG-CoA reductase was effectively decreased when compared to NLP-C rats (Ahmad and Beg 2013a; Ahmad and Beg 2013b). Reduced activity of HMG-CoA reductase was apparently due to involved in two related mechanisms: suppression of hepatic HMG-CoA reductase mRNA expression and enhanced low-density lipoproteins (LDL) receptor gene, as reported by Al-Naqeep et al. (2009) in rats treated with thymoquinone rich fraction. Such hypolipidmic regulation of these test fractions and pure compounds is apparently involved in reduction in lipid peroxidation. This hypothesis is also supported by our previous experiments that are significant mitigating role of administration of these test fractions and compounds in the reduction of CD and MDA products from LDL and its subfractions, sd-LDL and lb-LDL (Ahmad et al. 2013), and plasma was reported in atherogenic suspension fed rats (Ahmad and Beg 2013a; Ahmad and Beg 2013b; Ahmad and Beg 2013c).

From the above combined in vitro and in vivo studies, it was conferred that ME comprising TQ, LMN, linoleic acid with palitic acid called *compound triad* (Ahmad and Beg 2013a), is significantly effective in lipidemic oxidative stress in atherogenic suspension fed rats through hypolipidemic as well as free radical scavenging activities. The ME contained the highest quantity of linoleic acid (Ahmad and Beg 2013a); it was also supported by Ramadan and Morsel (2003) and Ramadan et al. (2003) where linoleic acid, an essential fatty acid was a dominating fatty acid in NS seeds. On the other hand, VO having thymol, thymoquinone and isothymol called *thyme triad* (Ahmad and Beg 2013a), being quite effective in amelioration of oxidative stress is deficient in essential fatty acid, linoleic acid and palmitic acid that show hypolipidemic property (Champe et al. 2008). VO also lacks the reduced form of limonene showing hypolipidemic effect (Ahmad and Beg 2013a). Thus, ME in comparison to VO showed strong antiperoxidative role in atherogenic suspension fed rats. However, all the above test fractions and pure compounds reduced the oxidative stress in rats and likely share common mechanisms i.e., suppression of HMG-CoA reductase activity and the increased level of LDL receptor (Al-Naqeep et al. 2009). As evident from in vitro antioxidant/free radical scavenging activities of these test fractions and pure compounds showed antiperoxidative or free radical scavenging activities in atherogenic suspension fed rats. Other active constituents present in ME and VO show independent or synergistic effect to mitigate lipidemic oxidative stress in rats. Thus, effective role of these test fractions and pure compounds were in the order of ME > TQ > VO > LMN in treated rats.

## Conclusion

In vitro ME, rich in phenolic as well as fatty acids compounds exhibited a higher antioxidant capacity than VO and TQ, while LMN was even a weaker radical scavenger than NS oil. Consistent with these results, in vivo investigation revealed that the test fractions and compounds, particularly, ME, effectively protected against lipidemic-oxidative stress-induced cellular damage of the erythrocyte and hepatic tissues, by preventing excessive lipid peroxidation. Thus, the combined results provide strong evidence in support of the use of above test fractions and compounds as free radicals scavenging/antiperoxidative agents. NS seed oil and its fractions, preferably, ME, may serve as excellent dietary source of natural antioxidants against variety of free radical attacks. So, these test fractions and compounds, preferably, ME, may also play as prophylactic or therapeutic roles against ROS/free radical mediated diseases e.g. cardiovascular disease, diabetes etc. In future, it is needed to find out scavenging/antiperoxidative properties of all chemical compounds present in both the test fractions to determine independent or synergistic effect of different identified compounds to elucidate their potential biological activities.
